# Secoisolariciresinol diglucoside regulates estrogen receptor expression to ameliorate OVX-induced osteoporosis

**DOI:** 10.1186/s13018-023-04284-5

**Published:** 2023-10-24

**Authors:** Guofang Chen, Yansong Chen, Junyi Hong, Junwei Gao, Zhikun Xu

**Affiliations:** Department of Orthopaedics, Zhejiang Xiaoshan Hospital, Hangzhou, 311200 Zhejiang Province China

**Keywords:** Postmenopausal osteoporosis, Secoisolariciresinol diglucoside, Ovariectomization, Estrogen receptor

## Abstract

**Objective:**

Secoisolariciresinol diglucoside (SDG) is a phytoestrogen that has been reported to improve postmenopausal osteoporosis (PMOP) caused by estrogen deficiency. In our work, we aimed to investigate the mechanism of SDG in regulating the expressions of ERs on PMOP model rats.

**Methods:**

Ovariectomization (OVX) was used to establish PMOP model in rats. The experiment was allocated to Sham, OVX, SDG and raloxifene (RLX) groups. After 12-week treatment, micro-CT was used to detect the transverse section of bone. Hematoxylin and Eosin staining and Safranine O-Fast Green staining were supplied to detect the femur pathological morphology of rats. Estradiol (E2), interleukin-6 (IL-6), bone formation and bone catabolism indexes in serum were detected using ELISA. Alkaline phosphatase (ALP) staining was used to detect the osteogenic ability of chondrocytes. Immunohistochemistry and Western blot were applied to detect the protein expressions of estrogen receptors (ERs) in the femur of rats.

**Results:**

Compared with the OVX group, micro-CT results showed SDG could lessen the injury of bone and improve femoral parameters, including bone mineral content (BMC) and bone mineral density (BMD). Pathological results showed SDG could reduce pathological injury of femur in OVX rats. Meanwhile, SDG decreased the level of IL-6 and regulated bone formation and bone catabolism indexes. Besides, SDG increased the level of E2 and conversed OVX-induced decreased the expression of ERα and ERβ.

**Conclusion:**

The treatment elicited by SDG in OVX rats was due to the reduction of injury and inflammation and improvement of bone formation index, via regulating the expression of E2 and ERs.

## Introduction

As a systemic bone disease, osteoporosis (OP) is characterized by decreased bone mass, damaged bone microarchitecture and increased bone fragility [1]. In developing countries, especially in Asia, the incidence of osteoporotic fracture appears to be on the rise [2]. Postmenopausal osteoporosis (PMOP) is one of the primary forms of OP. Its occurrence is closely related to the plummeting of estrogen level in postmenopausal women, which can cause problems such as accelerated bone turnover, destroyed bone microarchitecture, weakened bone strength and increased bone fragility and fracture risk [3, 4]. The decline in estrogen in the ovaries after menopause is an important cause of rapid bone loss and the early stages of OP in women [5]. Estrogen replacement therapy is commonly used in the clinical prevention and treatment of PMOP [6]. However, long-term use of estrogen will have endometrial hyperplasia, vaginal bleeding and other adverse reactions and even increase the risk of endometrial cancer or breast cancer [7, 8]. Several trials [9, 10] and several meta-analyses [11, 12] have indicated denosumab, pamidronate and zoledronate for the treatment of PMOP. In addition, the effects of biomarkers in the treatment of PMOP are also beginning to be investigated [13, 14]. In consequence, it is beneficial to explore more natural sources of estrogen for the treatment of PMOP.

Estrogen receptor (ER) is a ligand-activated nuclear transcription factor that regulates the action of 17-β estradiol (E2). ERs are abundant in the human body and are thought to be an important regulator of bone metabolism [15], with two subtypes, ERα and ERβ [16]. Estrogens bind to ERs to form dimers that bind to estrogen response elements on the genome, thereby regulating the transcription of estrogen-response genes and estrogen comes into play [17]. Estrogen plays an important role in bone growth, bone maturation and bone turnover to maintain bone metabolic balance [18]. Estrogen selectively activates intracellular signaling pathways depending on the receptor subtype to which it binds, and has a variety of biological activities. ERα is mainly expressed in the uterus, testis, pituitary gland, kidney, epididymis and adrenal gland, mammary gland, bone and some other target organs. ERβ is distributed in the ovary, prostate, testis, bone and other organs, but its affinity is different in different tissues due to the difference of ER [19]. PMOP reduces the level of estrogen, resulting in increased osteoclasts, bone resorption and bone loss leading to fractures. Research has shown that women should start taking estrogen at menopause and continue taking it indefinitely to prevent fractures [20].

Secoisolariciresinol diglucoside (SDG) is a phytoestrogen found in the mature seed of *Linum usitatissimum L.* which is similar to human estrogen [21]. As a phenolic ingredient in the herb, SDG has preventive effects on estrogen-dependent diseases such as breast cancer [22], prostate cancer [23], menstrual syndrome [24] and OP. The clinical efficacy of SDG in the prevention and treatment of PMOP in women has been confirmed, which could increase the serum calcium content and bone mass of patients, improve the sensitivity of bone to parathyroid hormone, promote the formation of new bone matrix and have significant effects on controlling bone loss [25]. SDG has also been reported to improve ovarian reserve function in aging mice by inhibiting oxidative stress [26]. In addition, SDG metabolites may play a direct role in flaxseed combined with low-dose estrogen therapy on OP in ovariectomized (OVX) rats [27]. However, the mechanism of how SDG affects the expression of ERs is not clear, and the effect of SDG compared with raloxifene (RLX), a drug commonly used in clinical practice for the treatment of PMOP, cannot be unified. In this study, the PMOP rat model was established by OVX. After the intervention of SDG and RLX, serum E2 was determined by ELISA, the pathological changes of bone tissue were detected by staining, and the expressions of ERα and ERβ were detected by immunohistochemistry (IHC) and Western blot to explore the mechanism of SDG on ERα and ERβ expression of ER on PMOP in castrated rats.

## Materials and methods

### Reagents and chemicals

SDG (IS0600) and RLX (IR0380) were supplied by Beijing Solarbio Technology Co., LTD (Beijing, China). Hematoxylin (H3136), Eosin (E4009) and Fast Green (F7252) were purchased from Sigma (USA). Rat E2 ELISA Kit (RX302856R), rat bone ALP ELISA Kit (RX300464R), rat CTX ELISA Kit (RX300268R) and rat NTX-1 ELISA Kit (RX301514R) were purchased from Ruixin Biotechnology Co., Ltd (Quanzhou, China). Rat PINP ELISA Kit (CSB-E1274r) was purchased from Wuhan Huamei Biological Engineering Co., LTD (Wuhan, China). BCIP/NBT Alkaline Phosphatase Color Development Kit (C3206) was purchased from Beyotime Biotechnology Co., LTD (Shanghai, China). Estrogen Receptor-alpha Antibody (AF6058), Estrogen Receptor-beta Antibody (AF6469), ERα Antibody (BF8047) and ERβ Antibody (AF6469) were purchased from Affinity (USA). β-actin Antibody (81115–1-RR) was purchased from Proteintech Group, Inc. (Chicago, USA). Goat Anti-Rabbit IgG H&L (HRP) was provided by Abcam (Cambridge, UK). Anti-rabbit IgG HRP-linked Antibody (7074) and Anti-mouse IgG HRP-linked Antibody (7076) were purchased from Cell Signaling Technology, Inc. (Danvers, MA, USA).

### Animals

SD rats were provided by Shanghai SLAC Laboratory Animal Co., Ltd under the animal license permit number SYXK (Zhe) 2021–0033. The rats were reared in a well-ventilated environment for adaptive feeding with a 12-h light/dark cycle at 23 ± 2 °C and 60 ± 5% humidity in the animal room of Zhejiang Eyoung Pharmaceutical Research and Development Co., Ltd for 7 days.

### Model establishment and administration

Twenty-four female non-pregnant SD rats (220 ± 20 g, 12 weeks) were randomly divided into four groups: Sham, OVX, SDG (30 mg/kg) and RLX (1 mg/kg). After one week of adaptive feeding, the groups of OVX, SDG and RLX underwent OVX surgery and the Sham group underwent sham surgery [28]. All animals received 3% pentobarbital sodium for anesthesia before the surgery. A longitudinal incision was made into the abdomen from both sides of the back, 1.5 cm beside the lumbar spine, and the ovaries were removed. Sham surgery performed a similar procedure but not carried out the step of ovariectomy. All rats received an intramuscular injection of penicillin (Hefei Dragon God Animals Pharmaceutical Co., Ltd.) once a day for three days. One week after surgery, the SDG group was given SDG (30 mg/kg) [29] by gavage twice a week, RLX group was administered RLX (1 mg/kg) [30] by intragastric administration twice a week, and the Sham group and the OVX group received the same volume of normal saline by intragastric administration for 12 weeks. The day after the last administration, the rats were respiratory anesthetized with 1.5% isoflurane, blood was immediately extracted from the abdominal aorta and allowed to stand for 30 min and then centrifuged at 3500r/min for 15 min, and the blood serum was collected and stored at − 80 °C. The rats were then killed by carbon dioxide euthanasia, and the femur tissue was isolated and stored at − 80 °C.

### Micro-computed tomography (Micro-CT)

The femurs of rats were scanned using a Micro-CT imaging system (Product model: MCT-III, ZKKS, China). Image acquisition and analysis of the femur followed the published guidelines for the micro-CT evaluation of rodent bones [31]. After the scanning was completed, the 3D image of the rat femur was reconstructed using the NRecon software. A fixed volume of interest was selected in the specimen center for histological data analysis. Variables measured included bone mineral content (BMC, mg), bone mineral density (BMD, mg/cc), bone volume fraction (BVF, BV/TV, %), trabecular number (Tb.N, 1 /mm), trabecular thickness (Tb.Th, mm) and trabecular separation (Tb.Sp, mm).

### Hematoxylin and Eosin (H&E) staining

The femur tissues were fixed in 4% paraformaldehyde. After dehydration, the femur tissues were embedded in paraffin wax. The femur paraffin blocks were then cut into 5-μm slides. The slides were de-waxed with xylene, washed with water and stained with hematoxylin and eosin. Afterwards, the femur tissues images were recorded at 200 × and 400 × magnification by using a microscopic imaging system (Product model: Nikon DS-Fi2, Nikon, Japan) with an optical microscope (Product model: Nikon Eclipse Ci-L, Nikon, Japan). A random number method was used to select five doctors from the pathological diagnostic physician database to score the staining. The scoring principles were as follows: 0 score: normal organizational structure; 1 score: slight injury of bone trabecular structure, increased degree of trabecular separation, pathological damage area < 25%; 2 scores: moderate damage to the trabecular structure, increased degree of trabecular separation, pathological damage area < 50%; 3 scores: severe damage to the trabecular structure of bone, disordered distribution of bone cells, pathological damage area < 75%; 4 scores: bone trabecular structure severely damaged, mature bone cells were severely reduced, and the pathological damage area > 75%.

### Safranine O-fast green staining

The femur tissues slides were dewaxed with xylene and gradient ethanol, washed with water and then stained with safranine and fast green. Images of femur tissues were recorded at 200 × and 400 × magnification under a microscopic imaging system (Product model: Nikon DS-U3, Nikon, Japan) combined with an optical microscope (Product model: Nikon Eclipse E100, Nikon, Japan).

#### ELISA

The levels of E2, interleukin-6 (IL-6), procollagen I N-terminal peptide (P1NP), alkaline phosphatase (ALP), C-terminal telopeptide of collagen type 1 (CTX-1) and cross-linked N-telopeptide of type 1 collagen (NTX-1) in serum were quantified using ELISA kits and referring to the manufacturer's instructions. All samples were repeated in duplicate, and the absorbance was detected spectrophotometrically using a CMaxPlus Microplate reader (Product model: Molecular Devices, USA) at 450 nm.

### ALP staining

The chondrocytes were derived from cartilages of the distal femur and proximal tibia of 5-day-old rats. The chondrocytes were digested with 0.2% type II collagenase at the condition of 37 °C for 8 h. For cell culture, the chondrocytes were inoculated at a density of 5.7 × 10^5^ cells/cm^2^, cultured in DMEM/F12 supplemented with 10% fetal bovine serum, 100U/ml penicillin and 0.1 mg/ml streptomycin and incubated in a cell incubator at the conditions of 37 °C, 5% CO_2_. Then ALP staining was performed, fixation with 4% paraformaldehyde, and ALP-positive osteoblasts were examined under a microscope (Product model: ICX41, Ningbo Shunyu Co., Ltd, China) to detect the ALP activity of the chondrocytes.

### Immunohistochemistry (IHC)

The femur slides were dewaxed with xylene, hydrated with graded ethanol series and water, inactivated with 3% H_2_O_2_ and antigen retrieved with EDTA. The slides were incubated with 5% BSA for blocking, followed by overnight incubation at 4 °C with primary antibodies for ERα and ERβ. The slides were washed with PBS and then incubated with the secondary antibody for 2 h at the condition of room temperature. Subsequently, the slides were coated with DBA for 5 min. The slides were counterstained with hematoxylin for 30 s and washed with water. Then, the slides were transparentized with graded ethanol series and dehydrated with xylene. Afterward, the slides were mounted under a microscopic imaging system (Product model: Nikon DS-U3, Nikon, Japan) with an optical microscope (Product model: Nikon Eclipse E100, Nikon, Japan).

### Western blot

The total protein of the bone tissue was extracted, and the protein concentration was measured using a BCA protein quantitative kit. The proteins were separated by SDS-PAGE, with 30 μg of protein loaded in each well under a voltage of 80 V-120 V, and transferred to a 0.45 μm PVDF membrane under an electric current of 200 mA for 2 h. The membranes were then blocked with 5% BSA for 1.5 h at room temperature and then incubated with primary antibodies of ERα and ERβ overnight at 4 °C. Membranes were incubated with the secondary antibody for 2 h at a condition of room temperature. Then, the peroxidase-coated bands were detected by ultra-signal ECL chemiluminescent solution, and the images were captured using a Chemidoc XRS Imaging System (Product model: 610,020-9Q, Shanghai Qinxiang Scientific Instrument Co., Ltd, China). Image-Pro Plus 6.0 was used to make the signal intensity expression of the protein bands of interest quantified and normalized to β-actin.

### Statistical analysis

Data results were processed using SPSS. If the measurement data among multiple groups followed the normal distribution and the homogeneity test of variance, one-way ANOVA analysis of variance was used. Tukey test was used for comparison between groups. Dunnett's T3 test or independent sample *t* test was used when the distribution was normal, but the variance was not uniform. Kruskal–Wallis H test is used when the distribution was not normal. All data were expressed as mean ± standard deviation (mean ± SD). Significance level *α* = 0.05. A value of *P* < 0.05 was considered statistically significant.

## Results

### Effect of SDG on femoral parameters on femoral parameters of femur in OVX rats

**Fig. 1:**
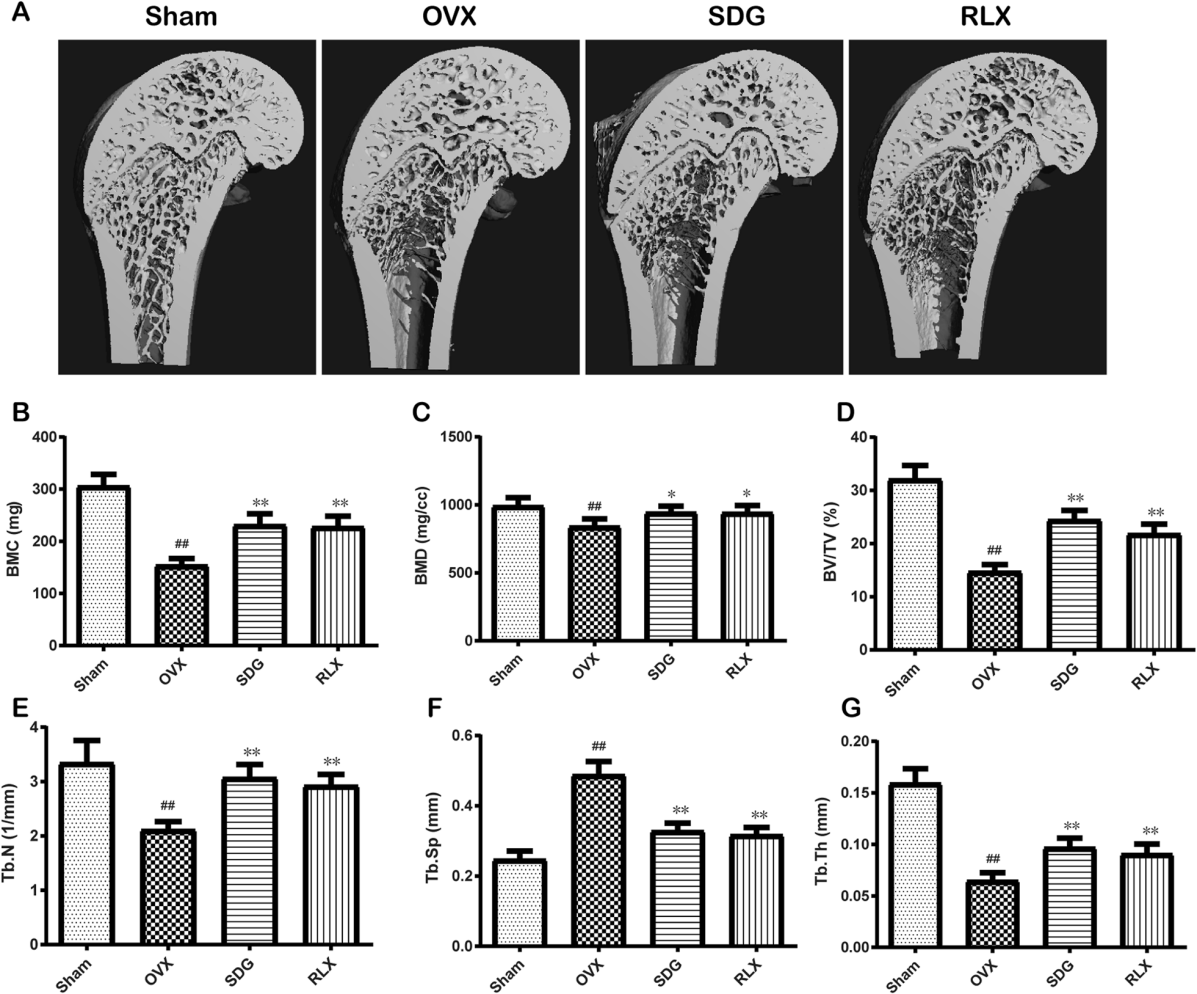
Effect of secoisolariciresinol diglucoside (SDG) on femoral parameters of femur in OVX rats. **A** The micro-CT images of the femur in rats. **B-G** The bone histological morphometric analysis results of the femur in rats, including bone mineral content (BMC), bone mineral density (BMD), bone volume fraction (BV/TV), trabecular number (Tb.N), trabecular thickness (Tb.Th) and trabecular separation (Tb.Sp). All data are expressed as the mean ± SD (n = 6). *P*^*##*^ < 0.01 vs. the Sham group, P^**^ < 0.01 vs. the OVX group

In Fig. 1A, compared with the Sham group, the micro-CT images showed that trabecular bone mass is decreasing significantly and the cancellous bone micro-architecture is deteriorating in the OVX group. The bone histological morphometric analysis (Fig. 1B-G) revealed that BMC, BMD, BV/TV, Tb.N and Tb.Th were decreased (*P*^*##*^ < 0.01) and Tb.Sp was increased significantly (*P*^*##*^ < 0.01) in the OVX group. SDG or RLX intervention could improve these parameters (P^*^ < 0.05 or P^**^ < 0.01).

### SDG reduced femur pathological damage and serum inflammation in OVX rats

**Fig. 2:**
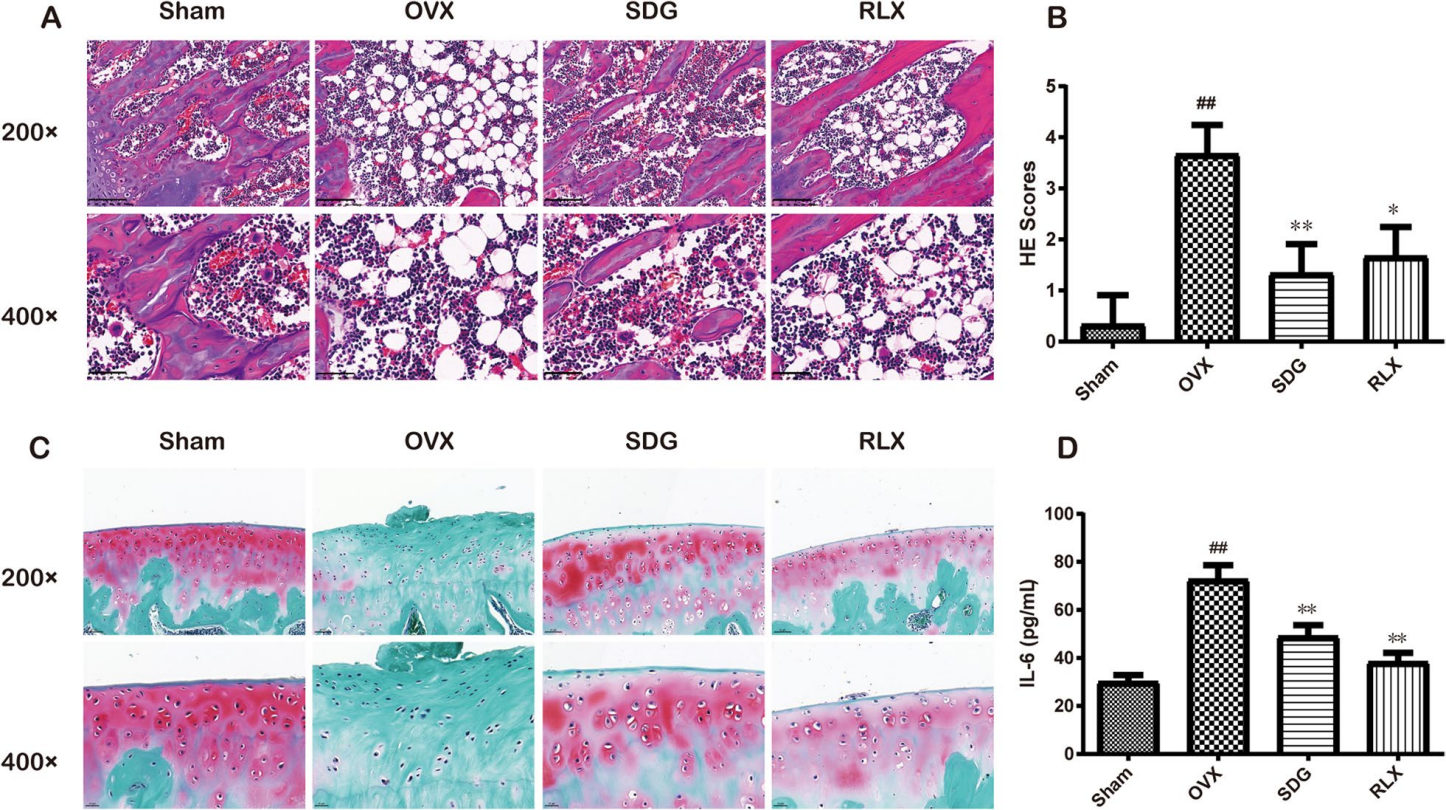
SDG reduced femur pathological damage and serum inflammation in OVX rats. **A** H&E staining results of the femur in rats (200 × , scale bar = 100 μm; 400 × , scale bar = 50 μm). **B** H&E staining score results of the femur in rats (*n* = 3). **C** Safranin and fast green staining results of the femur in rats (200 × , scale bar = 50 μm; 400 × , scale bar = 20 μm). **D** The serum level of IL-6 was detected by ELISA (*n* = 6). All data are expressed as the mean ± SD. *P*^*##*^ < 0.01 vs. the Sham group, P^*^ < 0.05, P^**^ < 0.01 vs. the OVX group

As shown in Fig. 2A, there was normal bone microarchitecture and neatly arranged bone trabecula in the Sham group, while in the OVX group, the femoral tissue was seriously damaged, the bone trabecula was sparsely arranged, the bone microarchitecture was severely damaged, and the mature bone cells were greatly reduced. Compared with the OVX group, SDG group and RLX group reduced the damage to the bone microarchitecture, the trabecular arrangement of bone was more regular, and the damage was less severe. In Fig. 2B, compared with the Sham group, the HE scores of femur tissue in the OVX group were increased significantly (*P*^*##*^ < 0.01). The HE scores of femur tissue decreased significantly in both SDG group (P^*^ < 0.05) and RLX group (P^**^ < 0.01) compared with OVX group.

Safranine O-Fast Green staining could detect cartilage tissue pathological changes. Basophilic cartilage is red when combined with Safranine O, and eosinophilic bone is blue when combined with Fast Green. From Fig. 2C, in the Sham group, the bone microarchitecture was normal with a smooth femoral surface, and there was no obvious injury. In the OVX group, the femur tissue showed large area loss of Safranin O staining, serious loss of proteoglycan, rough bone surface and severe damage of bone microarchitecture. Compared with the OVX group, the loss of Safranin O staining in femur tissue was reduced, the proteoglycan was partially lost, and the cartilage damage was attenuated in the SDG group and the RLX group.

ELISA results (Fig. 2D) showed that compared with the Sham group, the serum level of IL-6 in the OVX group was significantly increased (*P*^*##*^ < 0.01). SDG or RLX could decrease the level of IL-6 (P^**^ < 0.01).

### SDG regulated the indexes of bone formation and bone catabolism in OVX rats

**Fig. 3:**
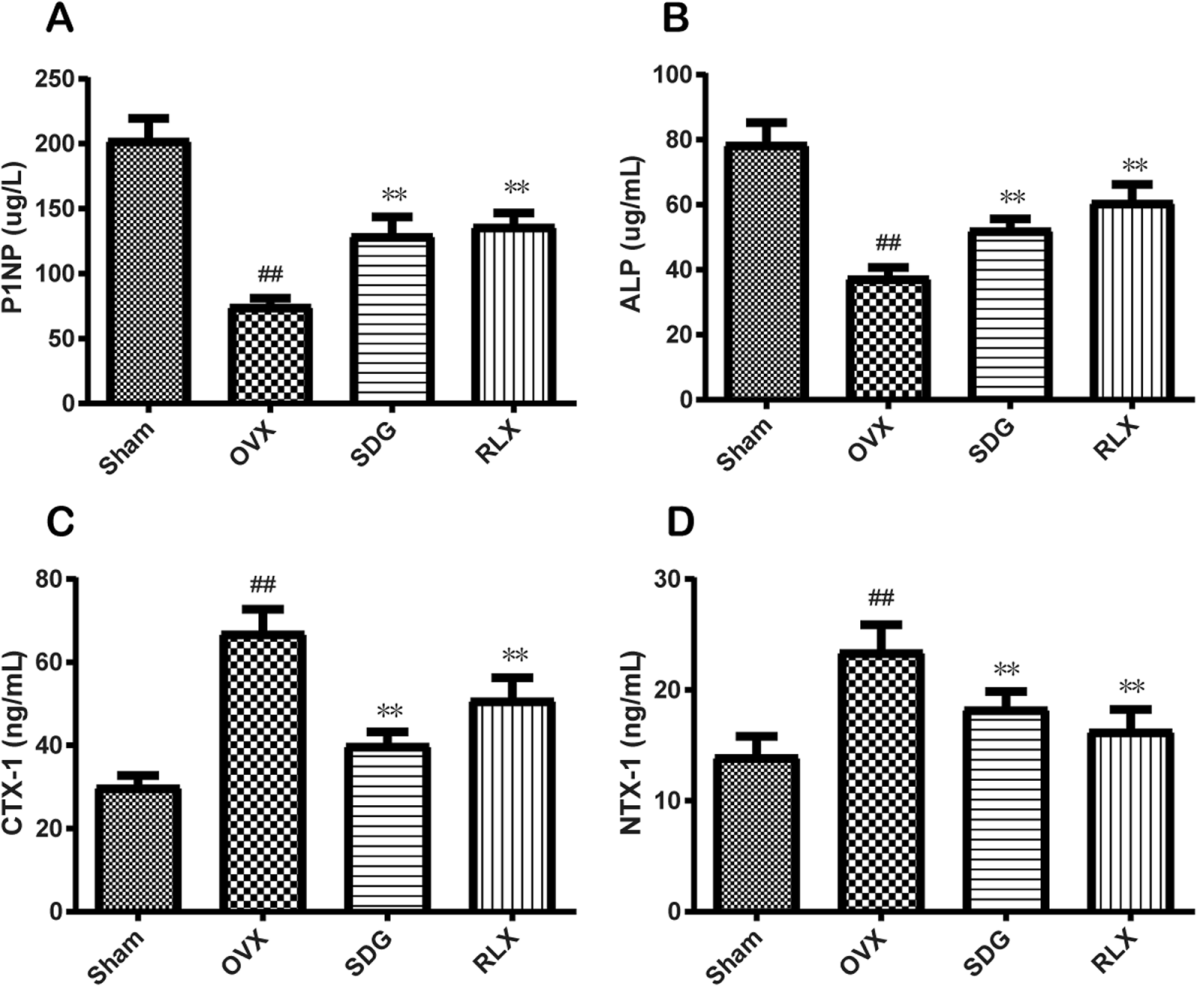
SDG regulated the indexes of bone formation and bone catabolism in OVX rats. **A-D** The serum level of PINP, ALP, CTX-1 and NTX-1 was detected by ELISA. All data are expressed as the mean ± SD (*n* = 6). *P*^*##*^ < 0.01 vs. the Sham group, P^**^ < 0.01 vs. the OVX group

ELISA results (Fig. 3A-D) showed that serum levels of P1NP, ALP in the OVX group were significantly decreased (*P*^*##*^ < 0.01) while CTX-1, and NTX-1 were significantly increased (*P*^*##*^ < 0.01). Compared with the OVX group, the levels of P1NP and ALP in the SDG group and RLX group were increased (P^**^ < 0.01), while CTX-1 and NTX-1 were decreased (P^**^ < 0.01).

### Effects of SDG on osteoblastic capacity of chondrocytes

**Fig. 4:**
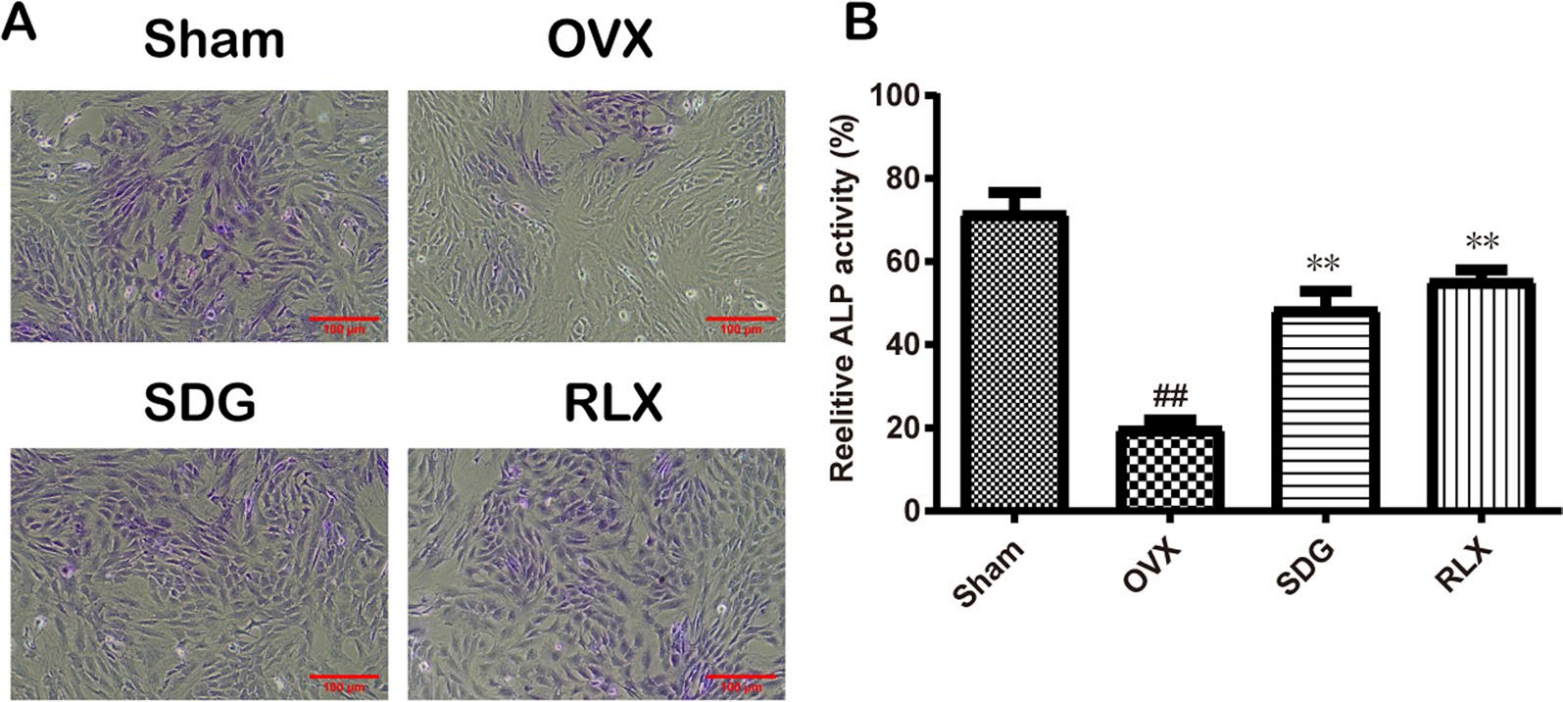
Effects of SDG on osteoblastic capacity of chondrocytes. **A** ALP staining results of chondrocytes (scale bar = 100 μm). **B** Statistical results of ALP staining degree of chondrocytes. All data are expressed as the mean ± SD (*n* = 3). *P*^*##*^ < 0.01 vs. the Sham group, P^**^ < 0.01 vs. the OVX group

The nuclei and cytoplasm of chondrocytes with positive ALP staining showed deep purple. In Fig. 4, compared with the Sham group, the degree of ALP staining of chondrocytes in the OVX group was significantly decreased (*P*^*##*^ < 0.01). Compared with the OVX group, the ALP staining degree of chondrocytes in the SDG group and RLX group was significantly increased (P^**^ < 0.01).

### SDG promoted the level of E2 and the expression of ERα and ERβ of the femur in OVX rats

**Fig. 5:**
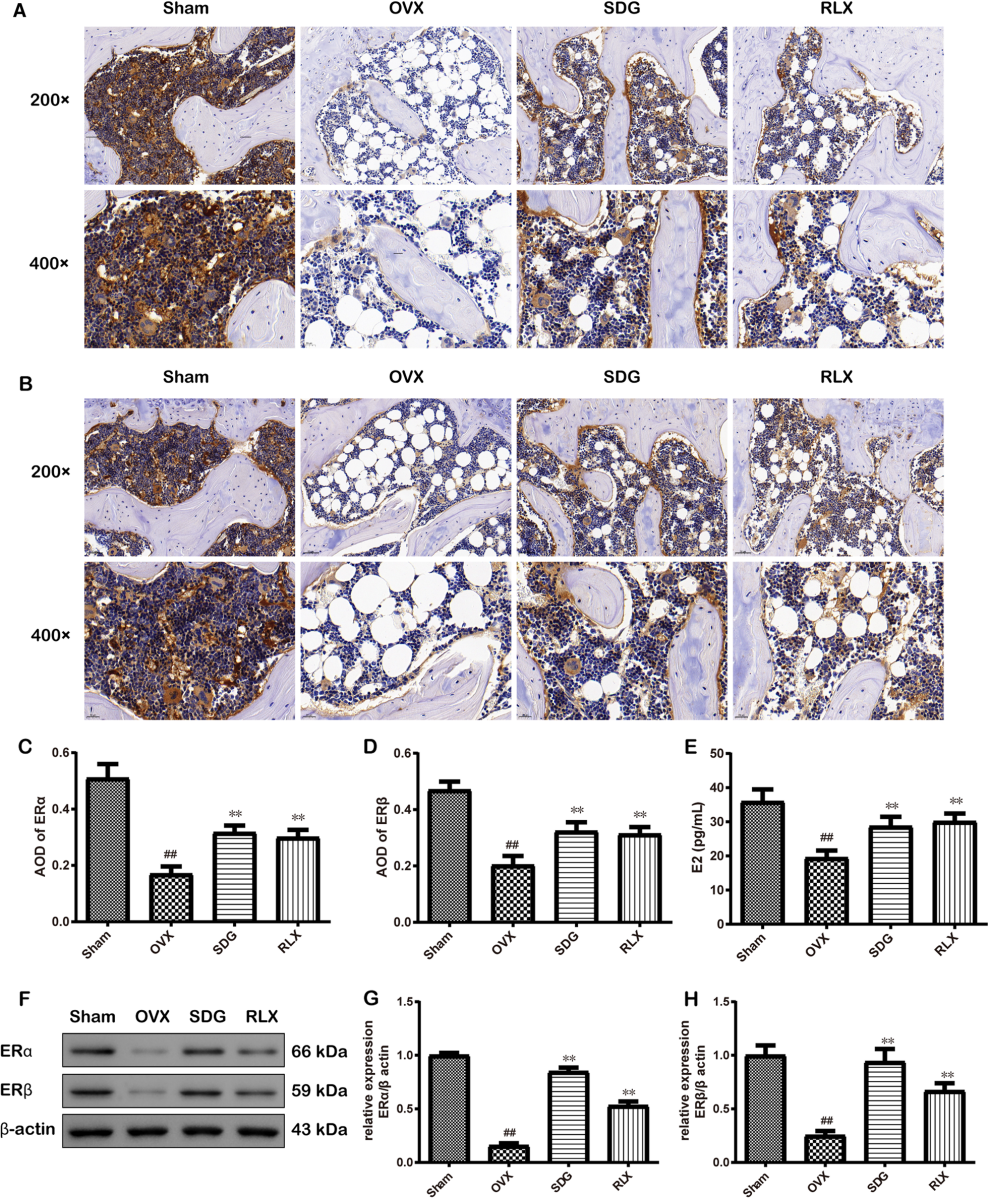
SDG promoted the level of estradiol (E2) and the expression of ERα and ERβ of the femur in OVX rats. **A-D** IHC result and quantitative statistical result of ERα (200 × , scale bar = 50 μm; 400 × , scale bar = 20 μm). **E** The serum level of E2 was detected by ELISA (*n* = 6). The expression of ERα and ERβ was determined by IHC and Western Blot. **F** Representative protein bands of ERα and ERβ. **G-H** The corresponding quantitative statistical results of ERα and ERβ (*n *= 3). All data are expressed as the mean ± SD. *P*^*##*^ < 0.01 vs. the Sham group, P^**^ < 0.01 vs. the OVX group

The results of IHC (Fig. 5A-D) showed that OVX caused lower expressions of ERα and ERβ (*P*^*##*^ < 0.01), while SDG or RLX treatment increased their expressions (P^**^ < 0.01). In Fig. 5E, the ELISA result showed that the serum level of E2 in the OVX group was decreased significantly (*P*^*##*^ < 0.01), and SDG or RLX treatment increased the level (P^**^ < 0.01). Western Blot (Fig. 5F-H) showed the same experimental results as IHC.

## Discussion

PMOP, as a disease caused by estrogen deficiency, occurs in postmenopausal women, women with a family history of OP or ovariectomy or premature menopause [32]. Current research into this disease is becoming increasingly detailed, and previous studies have found that estrogen plays a crucial role in the balance of bone remodeling [33]. Estrogen reduction leads to changes in the combination of estrogen and ER, which enhances osteoclast differentiation, decreases osteoblast proliferation and differentiation and increases cell apoptosis, and ultimately the capacity of bone resorption is greater than bone formation, and OP occurs [34]. In recent studies, researchers have focused on the treatment of OP caused by estrogen deficiency. Some studies have investigated the effects of Chinese herbal extracts or major constituents on the prevention and treatment of OP through OVX-estrogen deficient model rats [35]. SDG is a phytoestrogen very similar to human estrogens [36]. Clinical studies have shown that patients treated with SDG have significantly increased BMD and serum calcium, which enhanced the parathyroid hormone sensitivity and inhibited bone resorption, reducing the rate of bone conversion after treatment [37]. In addition, SDG could increase the weight of the femur, the content of serum calcium, serum phosphorus and bone calcium [38]. Bone histopathology studies showed that SDG could increase Tb.N, Tb.Th, BV/TV, increase the mean bone cortical thickness and reduce the mean Tb.Sp for the treatment of OP [39]. Therefore, we performed bilateral ovariectomy in SD rats [28] to establish an animal model of PMOP that reduce the estrogen levels. Consistent with previous research [40], in our study, the femur of rats that underwent OVX surgery demonstrated significant trabecular bone loss, decreased BMC, BMD, BV/TV, Tb.N and Tb.Th and increased Tb.Sp. We found that SDG treatment could significantly reduce OP via increasing BMC, BMD, BV/TV, Tb.N and Tb.Th and decreasing Tb.Sp, suggesting a substantial improvement in bone microarchitecture pathological damage.

Several studies have shown that almost all chronic diseases are associated with inflammation, including OP [41]. B cells and T cells, immune cells, are involved in the normal process of bone formation and bone resorption. The decrease in estrogen level after menopause led to the expansion of T cells and significantly increased contents of pro-inflammatory factors IL-1, IL-6, IL-17 and TNF-α. And then the function of osteoclasts was enhanced, and bone deposition capacity of osteoblasts was reduced, which made bone resorption greater than bone formation, leading to decreased bone mass and OP [42]. Our experimental results showed that OVX caused an increase in serum IL-6 content, which may imbalance the function of osteoclasts and osteoblasts, resulting in the occurrence of OP. IL-6 levels decreased after administration of SDG, suggesting that SDG ameliorated OP by reducing the expression of pro-inflammatory factors.

Bone turnover biomarkers (BTMs) are by-products produced during bone remodeling that can indicate the rate of bone turnover [43]. BTMs are mainly classified into bone formation markers and bone resorption markers. The specificity of these markers is limited, but BTMs levels show a clear and rapid response to changes in bone turnover rate, so BTMs levels are commonly used clinically to monitor treatment response and compliance in OP patients from the start of therapy [44]. Our study found that OVX reduced the contents of bone formation markers P1NP and ALP and increased the content of bone resorption markers CTX-1 and NTX-1, suggesting that OVX caused a decrease in bone formation and an increase in bone resorption. After the administration of SDG, the trend caused by OVX was reversed, suggesting that SDG could improve bone formation and reduce bone resorption, restore bone formation/resorption balance, and thus alleviate OP caused by PMOP.

Estrogen, a sex steroid, can directly enter the nucleus or cytoplasm of the target cell and bind to ERs. E2, as the major estrogen secreted by the female ovaries, can improve bone microstructure and BMD of the femur in PMOP rats, increase the content of bone formation indexes in serum and play a part in bone preservation [45]. As mentioned above, ERs play an important role in bone formation and bone resorption [34, 46]. Many researchers have reported that ERs influence BMD and PMOP [47]. The pharmacological experiments showed that SDG could affect hormonal parameters and increase the level of E2 in serum [48]. In this study, the experimental results showed that OVX decreased the content of E2 and the expressions of ERα and ERβ, indicating that the serum estrogen level and the expressions of ERs decreased after OVX surgery, leading to the occurrence of PMOP. After the administration of SDG, it was found that SDG significantly increased the E2 content and the expression of ERα and ERβ reduced by OVX, suggesting that SDG could inhibit PMOP by increasing the expressions of ERs.

Our current research still has some limitations. Although we have found that SDG could regulate the expression of ERα and ERβ, to treat PMOP, there is no specific mechanism between them. Further experiments are needed to clarify it, and there is still a long way to go before it can be used in the clinic. In the following studies, molecular docking, fluorescent probes and gene silencing will be effective methods to elucidate the mechanism in vivo and in vitro experiments.

## Conclusion

In conclusion, our results show that SDG has a therapeutic effect on PMOP model rats by regulating bone microarchitecture, reducing inflammatory damage and increasing bone formation markers, via activating the expression of ERα and ERβ.

## Data Availability

The data presented in this study are available upon request from the corresponding author.
